# Exploring the effect of automation failure on the human’s trustworthiness in human-agent teamwork

**DOI:** 10.3389/frobt.2023.1143723

**Published:** 2023-08-23

**Authors:** Carolina Centeio Jorge, Nikki H. Bouman, Catholijn M. Jonker, Myrthe L. Tielman

**Affiliations:** ^1^ Interactive Intelligence, Intelligent Systems Department, Delft University of Technology, Delft, Netherlands; ^2^ Leiden Institute of Advanced Computer Science (LIACS), University of Leiden, Leiden, Netherlands

**Keywords:** human-automation teamwork, automation failure, mental model, trust, trustworthiness, human-agent collaboration

## Abstract

**Introduction:** Collaboration in teams composed of both humans and automation has an interdependent nature, which demands calibrated trust among all the team members. For building suitable autonomous teammates, we need to study how trust and trustworthiness function in such teams. In particular, automation occasionally fails to do its job, which leads to a decrease in a human’s trust. Research has found interesting effects of such a reduction of trust on the human’s trustworthiness, i.e., human characteristics that make them more or less reliable. This paper investigates how automation failure in a human-automation collaborative scenario affects the human’s trust in the automation, as well as a human’s trustworthiness towards the automation.

**Methods:** We present a 2 × 2 mixed design experiment in which the participants perform a simulated task in a 2D grid-world, collaborating with an automation in a “moving-out” scenario. During the experiment, we measure the participants’ trustworthiness, trust, and liking regarding the automation, both subjectively and objectively.

**Results:** Our results show that automation failure negatively affects the human’s trustworthiness, as well as their trust in and liking of the automation.

**Discussion:** Learning the effects of automation failure in trust and trustworthiness can contribute to a better understanding of the nature and dynamics of trust in these teams and improving human-automation teamwork.

## 1 Introduction

Automation shows benefits for humans in terms of improved decision-making, performance, and reduced workload (Parasuraman et al., 2000), which is why it can be beneficial for humans and automation to collaborate. These collaborations result in human-automation teams, which are becoming increasingly common in life-saving situations (Laurent et al., 2019; Wei et al., 2018; Aggarwal, 2019). In such teams, trust between the teammates is essential for the successful functioning, since trust connects similar interests and pro-team behaviour, and creates behavioural norms that encourage collaboration (Groom and Nass, 2007).

Trust, however, is not a simple concept. Literature has focused on exploring trust in human-automation teams, particularly looking into the differences between human-human and human-automation trust (Alarcon et al., 2023; Eicher et al., 2023; Zhang et al., 2023), how this trust can be optimised (Lee and See, 2004; Groom and Nass, 2007; Webber, 2008; Knocton et al., 2023), and which factors reduce trust (Falcone and Castelfranchi, 2004; Madhavan et al., 2006; Kopp et al., 2023). In particular, automation failure has a significant impact on a person’s trust, i.e., a person that is interacting with the imperfectly reliable has a significantly lower level of trust in it in subsequent interactions (Robinette et al., 2017). Without sufficient trust, team members are less willing to be vulnerable and accept risks, which will decrease engagement in cooperation and consequently their reliability (Falcone and Castelfranchi, 2004; Salas et al., 2005; Tullberg, 2008). This means that when trust decreases due to automation failure, it may also mean that the human collaborator will be less willing to collaborate, thus less trustworthy in that interaction. However, in a study by Salem et al. describing a situation where the automation asks a person to perform a task, it is found that there might not be an influence of trust on the trustor’s trustworthiness (Salem et al., 2015). Therefore, in this paper, we aim to examine the influence of automation failure in a human-automation collaborative setting.

As an illustrative example, imagine both a robot and a human are in a teamwork scenario, collaborating on several tasks with different levels of interdependence. In such situation, the human collaborator may need to assist the robot once this calls for help. The low level of trust of the human teammate in the robot (after failure) may decrease the willingness of this human to offer help, prioritizing its own tasks first, for example, instead of opting for jointly actions. This decreases the human trustworthiness in this interaction, meaning the robot may not rely on the human teammate to be as helpful, in this example. Such information can be used to adapt its interaction, e.g., not depend so much on the human, find repairing strategies, etc., (Kox et al., 2021; Zhang et al., 2023). Further investigation on the dynamics of trust and trustworthiness in human-automation teamwork is crucial to ensure its effectiveness. As such, our main research question is “What is the effect of automation failure on the human’s trustworthiness in human-automation teamwork?” We also investigate the effect of automation failure on the human’s trust in and liking of the robot. Finally, we investigate the relationship between trust in the robot and human trustworthiness in human-automation teamwork.

This paper presents an online experiment in a 2D grid-world where a virtual robot and a human need to collaborate to succeed in a “moving-out” task, where packages have to be moved outside a house. Through this study, we explore the effect of automation failure on the human’s trustworthiness in a human-automation collaborative team. Thus, the main contributions of this paper are:

• An environment developed in MATRX for studying human-automation teamwork interaction with automation failure, involving tasks with different levels of interdependence.
• The collection of data for the differences in human trustworthiness, trust and liking of the robot, between regular human-automation teamwork and human-automation teamwork with automation failure.
• The analysis of the effect of automation failure in human trustworthiness, trust in and liking of the robot, as well as the relationship between trust and trustworthiness, in human-automation teamwork scenario.

This paper first discusses the background and related work to the research in Section 2, after which a methodology is introduced in Section 3 Finally, the results are presented in Section 4 and then discussed in Section 5, ending with a conclusion.

## 2 Background and related work

Trust is a social construct that originates from interpersonal relationships (Dagli, 2018). This paper defines trust as the willingness of a party (the “trustor”) to be vulnerable to the actions of another party (the “trustee”) (Mayer et al., 1995). With this, trust is based on the expectation that the trustee will perform a particular action important to the trustor, irrespective of the ability to monitor or control the trustee. This implies a situation in the trustor is vulnerable, and their vulnerability rests with the actions, behaviours, or motivations of the trustee (Wagner et al., 2018). Trust is a subjective attitude of the trustor, which involves the *perceived* trustworthiness of the trustee (Centeio Jorge et al., 2021).

On the other hand, trustworthiness can be seen as an objective property of the trustee. This paper follows the definition of Mayer et al., who define it as the extent to which an actor has the ability to execute relevant tasks, is benevolent towards its teammates, and demonstrates integrity (Mayer et al., 1995). Here, ability refers to the skills and knowledge that enable one to have influence within some specific domain. Benevolence is defined as the trustor’s belief in the trustee’s desire to do good on behalf of the trustor (wanting to help). Lastly, integrity is the trustor’s belief that the trustee adheres to a set of principles that the trustor finds acceptable.

Before the perception of trustworthiness of a trustee, trustors already have a likelihood to trust the trustee. This is called their propensity to trust. This can be thought of as a general willingness to trust others (Mayer et al., 1995). It influences how much trust the trustor will have in the trustee before the trustor knows the details of the trustee. In particular, the higher the trustor’s propensity to trust is, the higher the trust in a trustee is prior availability of information about this trustee (Mayer et al., 1995, p. 716).

### 2.1 Human-automation teams

In this paper we look at the effect of automation failure in the context of human-automation teams. A human-automation team is a team that consists of at least one human and one automation. In such teams, knowledge is shared, where the teammates depend on each other’s output, and work together on common functions (Chen and Barnes, 2014). In this paper we define automation as any sensing, detection, information-processing, decision-making, or control action that could be performed by humans but is actually performed by a machine (Moray et al., 2000, p. 1).

People interact with automation on a daily basis (e.g., a Google Assistant, self-driving car, or robot vacuum cleaner). Such automation is increasingly being developed as partners rather than tools (Klein et al., 2004), allowing humans to focus on their own tasks and strengths and covering their weaknesses. Successful technologies take advantage of such differences in strengths and weaknesses, as human reasoning has different characteristics than algorithmic reasoning (Chen and Barnes, 2014). For example, algorithms may only achieve limited accuracy, but they outperform humans because of their consistency (Kahneman and Klein, 2009), making them more suitable for tasks that are too repetitive, fast, or dangerous for humans to perform (Kohn et al., 2021).

To maintain credibility and performance in these teams, frequent interaction with the members of a team is considered as an important element of team effectiveness. This builds a relationship with the other members of the team, resulting in greater trust (Webber, 2008), and trust between teammates is essential for the successful functioning of a team (Groom and Nass, 2007).

### 2.2 The difference of trust in humans and automation

Human-human relationships are conceived differently from human-automation relationships, where an assessment of trust/distrust seems to be dependent on different factors, see, e.g., (Jian et al., 2000; Alarcon et al., 2023; Eicher et al., 2023; Zhang et al., 2023). Benevolence, for example, is about interpersonal relationships, meaning it might not develop in human-automation relationships the same way it does for human-human relationships (Centeio Jorge et al., 2021). Furthermore, there is symmetry to interpersonal trust, in which the trustor and trustee are each aware of the other’s behaviour, intents, and trust (Deutschi, 1960). However, there is no such symmetry in the trust between humans and automation (Lee and See, 2004). This makes it difficult for humans to trust something that is unable to trust and to feel guilt or betrayal in the same way (Groom and Nass, 2007). Moreover, it has been shown that the propensity to trust humans also differs from the propensity to trust automations (Hoff and Bashir, 2015).

Studies suggest that people perceive automation as a more credible source of information than humans (Lee and Moray, 1992; Wright et al., 2016). However, humans also tend to rely on their own decisions, even when provided with feedback that their performance was inferior to that of the automation (Dzindolet et al., 2002), where humans also tend to blame the automation for negative outcomes (Morgan, 1992; Frieainan, 1995), while being reluctant in giving credit to the automation (Madhavan and Wiegmann, 2007). The less a human trusts the automation, the sooner they will intervene in its progress of a task (Olsen and Goodrich, 2003). Therefore, human trust in automation depends on several factors, including the timing, consequences, and expectations associated with failures of the automation (Lee and See, 2004; Merritt et al., 2015).

### 2.3 The effect of automation failure

Research shows that a single error from automation strongly affects a person’s trust (Robinette et al., 2017), such that a mistake made by an automation will cause a person to have a significantly lower level of trust in it in subsequent interactions (Robinette et al., 2017). When humans have high expectations, there is a steeper decline in trust in case of an automation failure than it would in case of a human error (Madhavan et al., 2006). In other words, humans expect automation to have a near perfect performance, causing people to pay too much attention to errors made by automation (Dzindolet et al., 2002), whereas they do not expect their human partners to be perfect.

Automation failure reduces trust, and when the trustor has such reduced trust in the trustee, the trustor may also be less willing to be vulnerable and accept risks (Alarcon et al., 2021), which may decrease cooperation and reliability, thus reducing their own trustworthiness towards the trustee in that interaction (Falcone and Castelfranchi, 2004; Salas et al., 2005; Tullberg, 2008). This is found in human-human studies (Tullberg, 2008) or in multi-agent studies based on human-human theories (Falcone and Castelfranchi, 2004). This means that when trust decreases due to automation failure, it may also mean that the human collaborator will be less willing to collaborate, thus less trustworthy in that interaction.

A study in a human-automation non-collaborative setting suggests that a reduction in trust might not influence the trustor’s trustworthiness (Salem et al., 2015). However, this study found a significant difference in trust in their two conditions (one with automation failure and one without) with marginal results. Furthermore, the experiment design does not translate to different settings.

As such, in literature we find a decrease in human trustworthiness in human-human relationships, and no change in trustworthiness when the automation delegates the human, but there is no research on what happens in a human-automation collaborative setting. We conduct a study involving human-automation teamwork, aiming to fill the scientific gap on this part of the trust dynamics in human-automation teams.

## 3 Methodology

To test the effect of automation failure in human-automation teams, an experiment is conducted. This experiment examines the change in a human’s trustworthiness factors, as well as reported trust and liking of the robot, comparing participants who experienced automation failure with those who did not.

### 3.1 Hypothesis

In this paper, we hypothesise that the human’s trustworthiness decreases when automation failure occurs, as we propose that at least benevolence and integrity towards automation would significantly decrease if the automation fails to perform the collaborative task. This results into the following main hypothesis: Automation failure has a negative effect on the human’s trustworthiness in human-automation teamwork. We also hypothesise that automation failure has a negative effect on human’s trust in and liking of the robot. Furthermore, we want to investigate the relationship between participant’s trust in the robot and the participant’s trustworthiness towards the robot.

### 3.2 Design

The experiment has a 2 × 2 mixed experimental design, where the two independent variables are automation failure and game (two games played after each other, differing in time), and the dependent variables are the human’s trustworthiness, trust in and liking of the robot. All participants are assigned to one of the two experimental conditions: either one with automation failure (the experimental group), or one without (the control group). The participant performs a 2D simulated task, referred to as a game, on the computer, collaborating with an automation. This game is executed twice, with a questionnaire about trustworthiness, trust and liking after each game.

### 3.3 Participants

We recruited 54 participants, resulting into 27 participants per group. There were 21 men and 33 women. 44 participants were ranged between 18 and 29 years old, five were between 30 and 39 years old, three were between 50 and 59 years old, and two were between 60 and 69 years old. They reported on their gaming experience, where we had four daily gamers, ten weekly gamers, twelve monthly gamers, and 27 participants who do not play any games. Each participant signed an informed consent form before participating in the study, which was approved by the ethics committee of our institution (ID 2303).

Since we split the participants in two groups, it was important to balance the age, gender, and gaming experience across the conditions. Participants were assigned depending on their answers on these questions, balancing the groups during the experiment, and this balance was tested afterwards with positive results.

### 3.4 Materials

The experiment is programmed in MATRX^1^, which stands for Human-Agent Teaming Rapid Experimentation, and is a Python package designed for human-agent team research. It provides a basic user interface in a 2D grid-world with human controlled agents, autonomous agents, and the possibility of teams. This gives the developer a basic structure to implement their experiment in. For this thesis, the MATRX core version 2.1.2 is used. We run the experiment on a Windows computer with an Intel Core i7-6700HQ CPU 2.60 GHz processor and 8 GB RAM.

### 3.5 Task

The task is inspired by the game *Moving Out* ^2^ by DevM Games and SMG Studio. The goal of the task we designed in MATRX is to collaboratively move boxes to the correct location (in what we call the “dropzone”), all within the time restriction. There are two agents in the field: the human (controlled by the participant) and the automation (from here on called the ‘robot’). The boxes that are spread over the field are of three different types, which determines whether they can be carried alone or together. The team score increases for every box delivered correctly into the *dropzone*, receiving extra points for delivering the boxes in the given order. The experimental group will experience automation failure when they play the game for a second time, which is further explained in this section after all the game’s aspects and design choices are elaborated on.

#### 3.5.1 Boxes

There are three types of boxes in the field that can be lifted and moved: light, medium and heavy. The light box (recognisable by its green colour and small size) can be carried by one agent. The medium box (recognisable by its yellow colour and medium size) can be carried by either one or two agents. However, if an agent chooses to carry it alone, they will be walking thirty times slower than usual (chosen based on pilot observations). Lastly, there is a heavy box (recognisable by its red colour and big size), which can only be carried together. All boxes can break when placed incorrectly, indicated by dents in the box and a darker colour, which is discussed later in this section.

The decision for these types of boxes is made because we want to make the agents depend on each other as much as possible, highly favouring collaboration, which is also seen as a positive force (Jones and George, 1998). The medium box makes it possible for the human to stop the collaboration, if they want to, consequently lowering the human’s trustworthiness. This way, we can easily observe the human’s behaviour and intentions, resulting in the ability to study the human’s trustworthiness.

#### 3.5.2 The dropzone

**FIGURE 1:**
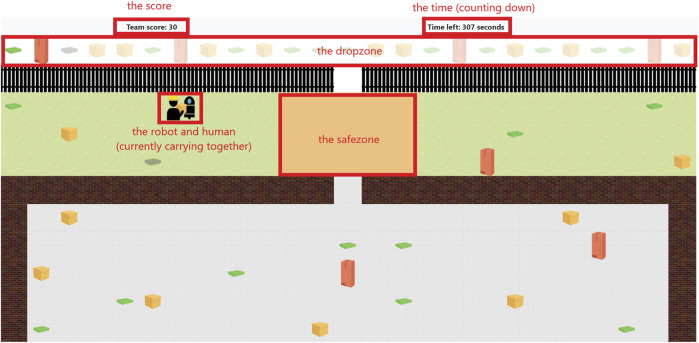
The game used in the experiment, programmed in MATRX. The red boxes and text is added in this work for explanatory purpose.

The dropzone is the line of more transparent boxes above the black fence, as can be seen in Figure 1. This is where the boxes in the field need to be delivered. When placing a box on the corresponding slightly transparent version, that box cannot be picked up again. When a box is placed outside the dropzone, it breaks, after which the first up box in the dropzone with that same type shows a broken image as well, indicating that box does not need to be delivered any more (Figure 1, the third box in the dropzone).

#### 3.5.3 The safezone

Boxes need to go to the dropzone, but it is possible for the human to accidently pick up a box that is not the next one in line. For this reason, the safezone was created. In this zone (indicated in orange, in between the wall and fence openings) boxes can be safely placed without breaking. All boxes that are placed outside the drop- or safezone break. The option to break boxes made for a way for an agent to deliberately break boxes, decreasing their trustworthiness.

#### 3.5.4 Agents

The field contains two agents: a human and a robot (Figure 1). The human is controlled by the participant with the ‘WASD’ or arrow keys, and can lift a box alone with ‘L,’ or call for help and lift the box together with the robot by pressing ‘H’. When an agent calls for help, a red exclamation mark appears next to the head of the agent, indicating the need for collaboration. A box is placed on the ground by pressing ‘P’.

The robot walks around autonomously, having an overview of the dropzone and all the boxes in the field. In short, the robot would go through the following steps:

1. Check the dropzone, which box is next in the order given in the dropzone?
2. Find the (closest) box of that type in the field.
3. Walk to that box.
4. (Ask for help with that box, depending on its type)
5. Carry the box (alone or together).
6. Walk to the corresponding place in the dropzone.
7. Place the box.

While the robot is carrying a box, it checks whether the next box according to the order in the dropzone has changed, since the human can be quicker, placing the same type of box on the desired location before the robot is able to. The robot also continuously checks what the human is carrying. If the robot discovers that it is carrying the same type of box as the human, it places the box in the safezone, trying to keep the collaboration as smooth as possible. Lastly, the robot constantly checks whether the human asks for help. If they do, and the robot is not currently carrying anything, it immediately goes to the human. If the robot is carrying something, it first places the box in the destination, and then goes to the human, if they are still asking for help. It is important to note that the details of the robot implementation were not shared with the participants before or during the experiment.

#### 3.5.5 Time

A time restriction is added to force the participant into making a decision to complete the task as quickly as possible. For example, the human will notice that the robot is failing to do their job, so because of time constraints, the human would not try to carry all the medium and heavy boxes with the robot, hoping that the robot will not drop them, but rather aim for the light boxes to be sure of the delivery. Aside from this reasoning of experimental design, the time restriction makes the experiment more convenient in practical terms, since people with less gaming experience could possibly take longer to finish the task.

#### 3.5.6 Score

The game keeps track of the team score. Each box that is correctly placed in the dropzone contributes ten points to the score, regardless of the type of box. Boxes can be placed in the dropzone in any sequence, but delivering them from left to right (without skipping any) gives the team five extra points per box. To ease the decision to stop the collaboration, all boxes add the same amount of points to the score. Moreover, the five extra points they gain for following the sequence nudges the human towards collaborating with the robot. Making boxes worth different points could make the extra points inconsiderable.

In this experiment, there are twenty-five boxes located in the dropzone (twelve light, eight medium, five heavy). When a box is broken, the extra five points can still be received for the box next to it. This means that the human can choose to purposely break a box, thus skipping it, without losing the extra points. This also creates a way to make it evident to see that the human’s trustworthiness has decreased, for example, if the human decides to only break heavy boxes.

To emphasise the concept of collaboration, the use of a team score is chosen rather than individual scores. The extra points awarded to the team for placing a box in the correct order is given to compel the participant to stick to the order. In other words, the extra points are given to force the user to lift all the types of boxes. Without forcing the order, there is no particular reason for the user not to carry all the green boxes on its own first.

### 3.6 Automation failures

If the participant is in the experimental group and currently playing the second game, then the robot has to show faulty behaviour. This failure should be a performance-related factor (e.g., reliability, false alarm rate, failure rate, etc.), since those were found to be better predictors of trust development than attribute-related factors (e.g., robot personality, anthropomorphism, etc.) (Hancock et al., 2011). Therefore, the focus was to let the robot fail in terms of their performance. This consists of breaking boxes, placing them in the wrong location in the dropzone, or picking up a box that is not the next up box according to the dropzone sequence.

Overall, the robot breaks eight boxes during the game (two light, four medium, two heavy). The emphasis lies on the medium boxes, since they can optionally be carried alone or together. Four boxes are delivered in the wrong place, which are always light boxes, since the robot is not in control when carrying the medium and heavy boxes. Lastly, three boxes are collected out of order. This can be any type of box, but if it is not a light box, the robot merely asks for help at the ‘wrong’ box.

### 3.7 Measurements

To observe how the human’s trustworthiness evolves when the automation fails, we need a way to measure their trustworthiness. We do this via a questionnaire^3^ (subjective measurements) and by observing the human’s behaviour (objective measurements). As we believe that trustworthiness may be related to the trust in the robot and the liking of the robot, we also include subjective reported measures on trust and liking.

#### 3.7.1 Trust in the automation

Asking the participant to self-report their own level of trust is extremely common within this field of research (Hancock et al., 2011). Many existing questionnaires to measure the perceived trustworthiness of another agent exist (e.g., Singh et al., 1993; Madsen and Gregor, 2000; Adams et al., 2003; Cahour and Forzy, 2009; Merritt, 2011). Several of these questionnaires are discussed and reviewed by Hoffman et al. (2018), where a final questionnaire is concluded, adapting many items from (Merritt, 2011). Since this author has more useable scales on other factors that we want to measure (which will be discussed in the next paragraphs), we decided to use her scale to measure the factors of perceived trustworthiness.

The trust scale is evaluated in an experiment in which participants had to use a fictitious automated weapon detector with the task to screen luggage. The Chronbach’s alpha ranged from *a* = 0.87 to *a* = 0.92. The participant could answer to the statements in a 5-point Likert-type response scale ranging from *strongly disagree* to *strongly agree*. The statements were stated from the human’s perspective, for example, focusing on whether the human thinks they could rely on the robot. Since this automation was used for advice, we have to alter the statements to fit the context of our task, changing it to the robot from our experiment and its ability to deliver boxes.

#### 3.7.2 Human trustworthiness

The most essential concept we want to measure is the *human’s own perceived trustworthiness*, as this is a significant aspect in our research question. To maintain consistency in the questionnaire, we decide to use the same scale as the factors of perceived trustworthiness. The only difference is the subject, shifting from the robot to the human. (e.g., “I have confidence in the actions of the robot” becomes “The robot was able to have confidence in my actions.”)

A ceiling effect was occurring during the pilot of this study. Remembering that not only Likert scales but also sliding scales were often used for self-reports (Kohn et al., 2021), we decided to change this scale to a slider, providing more granularity. Moreover, the statements were exaggerated (e.g., “The robot was able to have complete confidence in my actions”), making it less tempting to fully agree with the statement.

The downside of self-report measurements is that they require interruption of the task, or, if administered at the end of the task, subject to memory failures and the participant’s bias (Kohn et al., 2021). Furthermore, self-report results do not consistently and perfectly align with actual trust behaviour (Kohn et al., 2021). Since the human’s trustworthiness is the most important concept in our research, we want to verify the results with objective measurements. With this, we cannot acquire a trustworthiness level equal to reality, as there is only so much we can observe, but we can reason what it means to be trustworthy in this specific experiment.

Benevolence towards the robot shows that you want to help the robot, and is one of the three factors of trustworthiness. In this experiment, wanting to help the robot can be observed by counting how many times the human would respond to the call for help from the robot. We will log:

• Participant answered to request for help from the robot: this may mean whether the participant is willing to help the robot.

Cooperation with the robot is another factor that shows trustworthiness and can be observed in this experiment. Being cooperative here means that medium and heavy boxes should be carried together without breaking, calls for help should be answered with actions of helping, and the participant should ask for help as well. For this, we will add to the log:

• Participant asked for help: this can show willingness to collaborate with the robot.
• Participant broke a box: As mentioned before in 3.5.6, when a box is broken, the participant can move on to the following box, without losing extra points. If the participant decides to break heavy boxes (which need to be carried jointly with the robot), this may mean that they are unwilling to collaborate with the robot.
• Participant carried a box alone: in the case of medium boxes, if the participant prefers to carry it alone, even though that means that option would take more time, it may mean the participant is unwilling to collaborate with the robot.
• Participant and robot carried a box together: on the other hand, carrying medium boxes together may show more willingness to collaborate.

The types of boxes are also registered with each action, making a distinction between carrying a medium or a heavy box together. These objective measurements allow for a comparison of the behaviours in the first and second game.

We wish to observe the ability of the participant. The game keeps track of the score, and logs it. However, this cannot provide us with an indication of the participant’s ability, since it is the collaborative score. When the participant is in the experimental group, the robot is manipulating this score, influencing the total score. Although the robot would want to break the same boxes in every experiment, it would depend on the participant on whether this box would actually be broken. For example, if the human always carries medium boxes alone, the robot would not be able to break a single medium box. We therefore decide to not include the participant’s ability when observing the objective trustworthiness.

#### 3.7.3 Propensity to trust

The author who developed the trust scale that was mentioned in the preceding paragraphs, has also constructed a propensity to trust scale (Merritt et al., 2013), which we also included in the questionnaire mentioned in the beginning of the section (available online). This scale contains questions concerning how likely the participant is to trust an automation without knowing the details of the automation. The participant can answer in a 5-point Likert-type response scale ranging from strongly disagree to strongly agree. We did not alter any questions from this scale.

#### 3.7.4 Liking the automation

The author of all the scales that we are using has developed a third scale that measurements liking (Merritt, 2011). If we would not include this scale, it would be the only part of the author’s questionnaire that we are not including. We therefore decide to include the liking scale in the experiment. This scale contains statements about the human’s feelings towards the automation (e.g., wishing the robot was not around) which could be answered in a 5-point Likert-type response scale. It is slightly altered to fit the context of our task (changing the automation in the questions to ‘the robot’).

#### 3.7.5 Strategy

A factor that was added to the questionnaire is the *strategy* of the participant. Knowing their strategy gives more insight into the decisions they made and possibly why their trustworthiness does or does not change. For example, a study found that participants developed a preference for less demanding tasks (Botvinick and Rosen, 2009). If such a thing is the case in our experiment, it would be convenient to know and take into account with the analysis. Moreover, by letting the participant read these possible strategies after the first game, they often realise what is actually possible during the game (e.g., during a pilot one of the participants said to understand why boxes can be broken, after reading the strategy about skipping boxes without losing the extra points). This will stimulate them to think about their actions, and make faster decisions if they encounter automation failure.

### 3.8 Procedure

After signing the informed consent, the participant would answer questions on their age, gender, gaming experience, and propensity to trust automation. Then they follow a tutorial for the game, after which they start the first game. Upon completion, they are asked about their trust in the robot, liking of the robot, own perceived trustworthiness, and their strategy. The participant then enters the second game, where they experience automation failure if they are in the experimental group. Afterwards, they are again asked about their trust in the robot, liking of the robot, own perceived trustworthiness, and strategy, where they can also state why they changed their strategy.

## 4 Results

This section reports the results of the experiment. We evaluated the effects of scenario on several measurements, including reported subjective trust, trustworthiness, and like scores, as well as objective measures that can show a participant’s trustworthiness, i.e., interactions with the robot and with the game. In particular, we studied the following objective measurements:

• Call for help: Number of times a participant called for the robot’s help.
• Response time to help: During the game, the robot calls the human for help with carrying a medium or heavy box. It is then for the human to decide how they respond to this. They could walk to the robot and carry the box together, or, in case of a medium box, decide to carry it alone, or even completely ignore the call for help. We define their response to help in seconds, counting how long it takes them to respond to the call for help. If they are carrying a box at the moment of the call, the timer will start as soon as they drop that box.
• Carrying boxes: We kept track of how many times the participant would carry a box with the robot compared to how many times they would carry a box alone. We divide the amount of times they carried together by the amount of times the participant carried alone. If this number is above one, the participant mostly carried boxes together, while if it is below one, the participant would mostly carry boxes alone.
• Breaking boxes The game is built around the option to break boxes. This is designed so that the robot can clearly show that it is less trustworthy. With this, we expected that the participant would then also break boxes, skipping the heavy boxes, while still receiving extra points for the order. However, during the game it quickly becomes clear that the participants do not like to break boxes, even during the tutorial. Whenever a participant does break a box, it is in the first game, and merely because they forgot the rule of the safezone.

**FIGURE 2:**
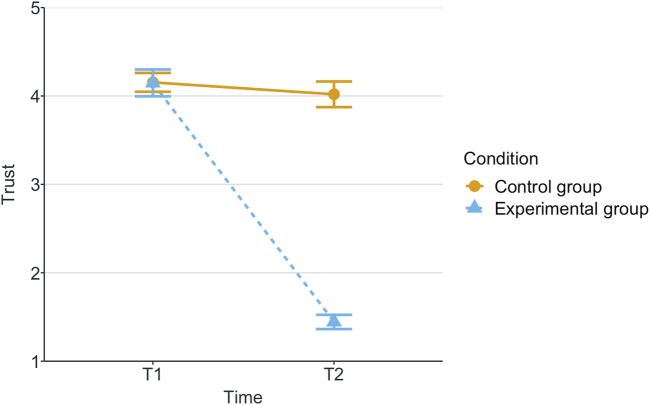
Mean of trust for each game and scenario.

**FIGURE 3:**
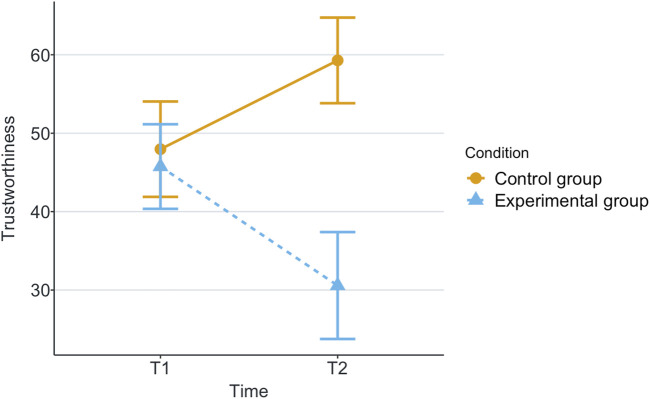
Mean of subjective trustworthiness for each game and scenario.

**FIGURE 4:**
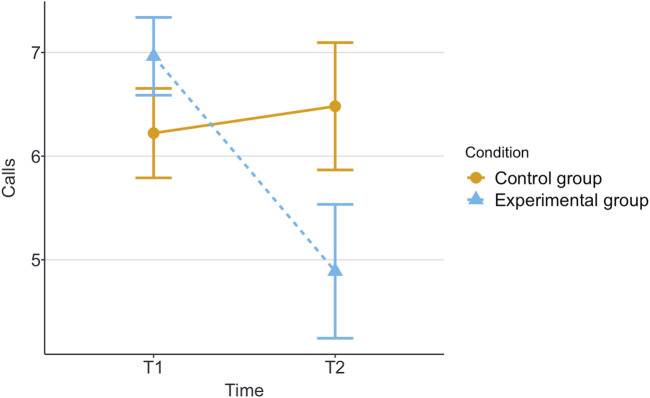
Mean of participant’s calls for robot’s help in each game for each scenario.

**FIGURE 5:**
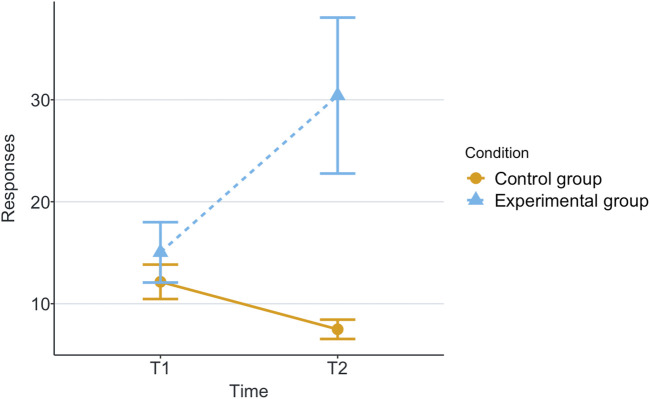
Mean of participant response time to robot calls in each game for each scenario.

**FIGURE 6:**
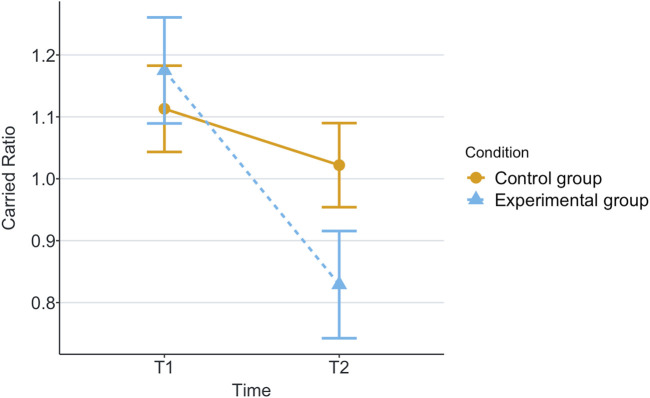
Mean of carried boxes in each game for each scenario.

**FIGURE 7:**
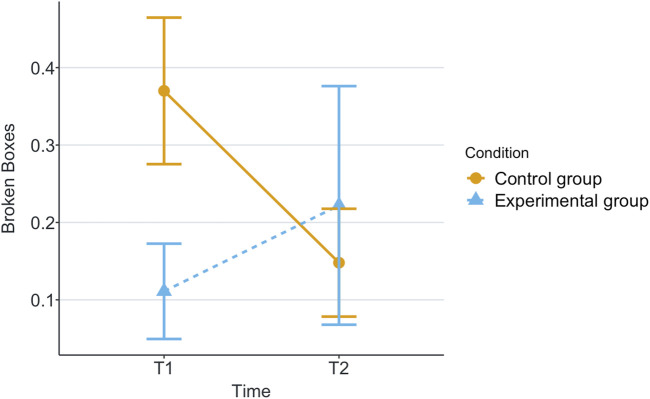
Mean of broken boxes by participant in each game for each scenario.

**FIGURE 8:**
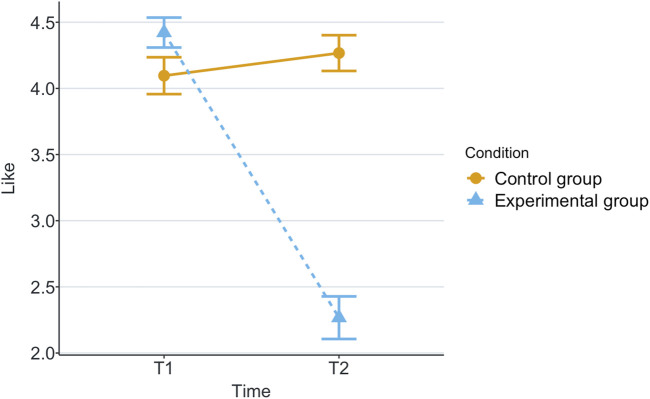
Mean like score at the end of each game in the two scenarios.

Table 1 shows the means and standard deviations (SD) per scenario (Group) and time for each of the measurements being evaluated. T1 corresponds to the end of the first game and T2 corresponds to the end of the second game. These values are plotted per measurement in Figures 2–8, where the solid and dashed lines show the change in means of control and experimental group, respectively.

**TABLE 1:** Mean and standard deviation (SD) of each measurement, per group and per time. These values are plotted in Figures 2–8.

Group	Time	Measurement	Mean	SD
Control	T1	Trust score	4.154	0.553
Experimental	T1	Trust score	4.148	0.788
Control	T2	Trust score	4.019	0.754
Experimental	T2	Trust score	1.444	0.419
Control	T1	Trustworthiness score	47.963	31.599
Experimental	T1	Trustworthiness score	45.747	28.026
Control	T2	Trustworthiness score	59.284	28.373
Experimental	T2	Trustworthiness score	30.574	35.414
Control	T1	Calls for help	6.222	2.242
Experimental	T1	Calls for help	6.963	1.951
Control	T2	Calls for help	6.481	3.191
Experimental	T2	Calls for help	4.889	3.355
Control	T1	Response time to help	12.149	8.774
Experimental	T1	Response time to help	15.036	15.379
Control	T2	Response time to help	7.490	4.929
Experimental	T2	Response time to help	30.416	39.734
Control	T1	Carried boxes ratio	1.113	0.362
Experimental	T1	Carried boxes ratio	1.175	0.445
Control	T2	Carried boxes ratio	1.022	0.353
Experimental	T2	Carried boxes ratio	0.829	0.450
Control	T1	Broken boxes	0.370	0.492
Experimental	T1	Broken boxes	0.111	0.320
Control	T2	Broken boxes	0.148	0.362
Experimental	T2	Broken boxes	0.222	0.801
Control	T1	Like score	4.096	0.724
Experimental	T1	Like score	4.422	0.588
Control	T2	Like score	4.267	0.702
Experimental	T2	Like score	2.267	0.836

For the analysis, we calculated the statistical significance of the scenario’s effects on the measurements with robust 2 × 2 mixed ANOVA, from the R package WRS2 (Mair and Wilcox, 2020). We have also calculated the effect size with a robust Cohen’s *d* (Algina et al., 2005), present in the same package. These effects can be found in Table 2. Reported subjective scores of trust, trustworthiness and liking (like) showed statistically significant effect among scenarios with effect sizes of large, small and medium, respectively. Regarding the objective measurements, only the participant’s time of response to robot’s calls for help had a statistically significant small effect size.

**TABLE 2:** Report of effect among scenarios calculated with robust 2 × 2 mixed ANOVA (Mair and Wilcox, 2020), and Cohen’s *d* effect size and interpretation based on (Algina et al., 2005).

Measurement	Robust 2 × 2 ANOVA	*p*-value	Effect size	Interpretation
Trust score	*F* (61.66, 1) = 29.65	<0.001 *	0.91	Large effect
Trustworthiness score	*F* (4.82, 1) = 31.98	0.04 *	0.44	Small effect
Calls for help	*F* (0.63, 1) = 30.64	0.4	NA	NA
Response time to help	*F* (4.61, 1) = 22.73	0.04 *	−0.45	Small effect
Carried Boxes Ratio	*F* (0.98, 1) = 30.66	0.33	NA	NA
Broken Boxes	*F* (3.74, 1) = 16	0.07	NA	NA
Like score	*F* (18.58, 1) = 27.42	<0.001 *	0.60	Medium effect

### 4.1 Strategy

The end of each part of the questionnaire contains a question about the participant’s strategy. They can tick off which strategy they were following, where multiple answers are possible. By analysing the histograms that result from the answers after the first game, split per condition, we observe that there is no notable difference when comparing the participants from the control group with those from the experimental group.

When looking at the answers after the second game, we observe a change in strategy in both conditions. We again observe very few people in either group has a strategy that involve breaking boxes. However, their way of carrying and delivering boxes does change. In both groups, there is an increase for carrying medium boxes alone, but we observe a much larger increase in the experimental group. Moreover, participants from the control group generally use the same strategy regarding the order of delivery, while participants from the experimental group change their strategy from delivery in the correct order to delivery in a random order. Another noticeable change is the increase of the amount of participants deciding to deliver boxes that can be carried alone first. In the control group, this is doubled, while in the experimental group the amount of people going for that strategy has become five times as much. With this delivery, there is an increase for delivering the closest boxes first for only the participants in the control group. Lastly, both groups show an increase for trying to carry a light box before the robot does it, but the increase in the experimental group was greater.

Ending the questionnaire, participants can indicate why they had changed their strategy. Most participants from the control group usually report that they had better knowledge of the game or the way the robot thinks, making this change in strategy a choice based on the score they want to obtain. Twenty-two participants from the experimental group report issues with the performance of the robot and their trust in the robot. Two state that they only changed their strategy because they were not able to get the high-score in the previous game, and three people did not answer the question.

### 4.2 Correlations

Using Linear Pearson correlation tests, we see moderate linearity for trust correlated with trustworthiness (*r* (52) = .49, *p* < .001).

## 5 Discussion and conclusion

The interpretation of our results will be elaborated around the main research question: What is the effect of automation failure on the human’s trustworthiness in human-automation teamwork?

### 5.1 Trustworthiness

Our research question was about finding an effect of automation failure on the human’s trustworthiness. Regarding the results, we confirm our hypothesis, stating that automation failure has a negative effect on the human’s trustworthiness in this study, Tables 1, 2. In particular, the reported subjective trustworthiness (trustworthiness score, in Figure 3) and objective trustworthiness-related metric of responding to robot’s calls for help (response time to help, in Figure 5) were negatively effected by automation failure (small effect size). Specifically, the trustworthiness score decreased after failure and the time to respond increased (showing less urgency to collaborate). Other trustworthiness-related metrics such as carried boxes (Figure 6), broken boxes (Figure 7), and calls for help (Figure 4) also show trends of possible effects of automation failure, but they were unfortunately not significant. However, this is worth exploring in other scenarios.

Our results align with the study of Tullberg and Falcone and Castelfranchi. Salem et al. stated that there might not be an influence of automation failure on trustworthiness, which can still hold, depending on the definition and degree of automation failure.

The findings seem to indicate that the human becomes less trustworthy when the automation starts failing, negatively affecting the collaboration between the two agents, thus negatively affecting the results. This knowledge is important, as it allows us to anticipate on the negative effects by, for example, having the robot apologising or explaining, as in Zhang et al. (2023); Kox et al. (2021).

### 5.2 Trust

As stated in Section 2.3, literature reports that one person’s trust in another affects their directed trustworthiness. For this reason, we evaluated whether the automation failure affected trust negatively. The results show a large effect size of automation failure in the trust score, see Figure 2 and Table 2. This is in line with previous research (e.g., Falcone and Castelfranchi, 2004; Madhavan and Wiegmann, 2007; Robinette et al., 2017). We see that, in this human-automation collaborative setting, a change in trustworthiness of the automation affects the trust that the human has in the automation. Moreover, we see a positive correlation between trust and trustworthiness scores in Section 4.2. Based on these results and the previously mentioned literature, we speculate that this decrease of participant’s trust in the automation then affects the participant’s trustworthiness towards automation. The causality effect of trust and trustworthiness in human-automation teams is worth further exploration in future work.

### 5.3 Liking

The results have shown that there was a medium effect size of automation failure in like score. This is a logical outcome, as liking is highly related to trust (Nicholson et al., 2001; Merritt, 2011).

### 5.4 Limitations

In the course of this research, we stumbled upon a few limitations. For example, the task design and decisions regarding the types of failures were several times arbitrary and could have an impact on these results. These include the time increment on carrying a medium box alone instead of jointly, for example, or the number of boxes the robot would break or place incorrectly. It should be noted that some of these decisions may have impacted the human’s perception of the robot’s performance and, therefore, reduce their trust and trustworthiness.

Furthermore, the ability of the participant could have been observed more closely, providing us with another indicator of their objective trustworthiness. We kept track of the game scores and whether the participant was carrying the box alone or together, but by making some kind of division for the team score to individual scores, we are still not anticipating the effect of the automation failure, or fully grasping the participant’s ability. For example, if we would give individual scores to the agents by observation (shared when they worked together, or individual points when one worked alone) and the participant would decide to work alone, they could potentially score more points in the second game than in the first game because the points are not shared, while they are not necessarily more capable than in the first game. This needs to be thought through, creating a solution for this experiment or one that involves a different type of experiment.

Moreover, it sometimes became clear that the participant did not understand every rule of the game. This did not happen often enough to discard the work, and it was not always the same rule that was forgotten (e.g., some participants forgot that a box would break, some forgot the effect of a broken box, some did not understand the rules of delivering in a certain order in combination with breaking boxes). Since they would understand after the first game, this could have affected the participant’s behaviour and thus the objective results from the second game. This could have been avoided by a longer tutorial, where they could participate in the game more independently. We expect that they would stumble upon their misinterpretations of the rules during this independent game, while not yet establishing an opinion about the robot, since it can be left out for this part. Another solution would have been to do a knowledge check on the rules. This would show their knowledge on the aspects of the experiment that could not have been observed by the instructor (e.g., the instructor might think that the participant knows the rule about the order of the boxes by their behaviour, but that is just a coincidence).

Lastly, what we measured as an increase in objective trustworthiness, could just be a choice of efficiency. For example, in both groups participants decided to carry light boxes first, and trying to get to them before the robot does. Participants from the control group reported that they did this to get a higher score. This is understandable when we consider that most participants were quicker than the robot. Participants from the experimental group reported that they decided to do this because they did not trust the robot to safely deliver it. Although the reasoning makes the division clear, such a division would be clearer in a group where a change in strategy for efficiency would lead to other participant behaviour than a change in strategy because of a decrease in trust. This should have been considered in the design of the experiment.

### 5.5 Future work

In the future of this research, it would be interesting to see the causality between trustworthiness, trust, and liking, as we can now only hypothesise. For example, we can raise the question whether the trustworthiness decreases because of the decrease in trust, or because of a decrease in liking. We do not know which of these factors affect which.

Moreover, we do not know whether all components of trustworthiness decrease. For example, it is possible that the participant’s ability increases, while their benevolence and integrity decreases. Knowing this, we could not only improve human-automation teamwork, but also use this information for the better of the participant (e.g., intentional automation failure to increase ability).

Lastly, we are curious to see which types of automation failure (e.g., false alarms compared to misses) have a larger effect on the human’s trustworthiness. This could involve other contexts, for example, a more serious context like a self-driving car. Knowing the degree of effect of such failures and other contexts does not only extend our knowledge of trust in human-automation teamwork, but could let us anticipate on the effects if necessary, or improve a study for repair strategies.

### 5.6 Conclusion

This study investigated how automation failure in a human-automation collaborative scenario affects the human’s trust in the automation, as well as a human’s trustworthiness towards the automation, which is not yet present in literature to the best of the authors’ knowledge. We presented a 2 × 2 mixed designed experiment in which the participants perform a simulated task in a 2D grid-world, collaborating with an automation in a “moving-out” scenario. During this experiment, we measure the participants’ trustworthiness, trust and liking regarding the automation both subjectively and objectively. The results show that automation failure negatively affects the human’s trustworthiness (both subjectively and objectively), and raises the question whether all factors of trustworthiness are affected, and whether all types of automation failures have this effect. This research shows relevant findings of previous research, helping to close the gap between human-human research and human-automation non-collaborative research, contributing to a better understanding of the nature and dynamics of trust in human-automation teams, and the possibility to foresee undesirable consequences and improve human-automation teamwork.

## Data Availability

The datasets presented in this study can be found in online repositories. The names of the repository/repositories and accession number(s) can be found in the article/Supplementary Material.

## Footnotes

1 matrx-software.com

2
https://www.team17.com/games/moving-out/

3 The questionnaire used can be found in https://repository.tudelft.nl/record/uuid:012a2cdb-4aa7-4667-b95d-02ae0baa6cde
