# Supplementary material for: Exploring the effect of automation failure on the human’s trustworthiness in human-agent teamwork
Source: Front Robot AI. 2023 Aug 23;10:1143723. doi: 10.3389/frobt.2023.1143723 (PMC10482046; doi:10.3389/frobt.2023.1143723)
Supplement: Supplementary file 1 [file DataSheet1.ZIP › moving out game/msc.-project-nikki-bouman-7-tutorial/moving_out/case/gui/templates/god.html]

God view


##### Chats

+

Action 1
Action 2
