# Supplementary figures and images for: Exploring the effect of automation failure on the human’s trustworthiness in human-agent teamwork
Source: Front Robot AI. 2023 Aug 23;10:1143723. doi: 10.3389/frobt.2023.1143723 (PMC10482046; doi:10.3389/frobt.2023.1143723)

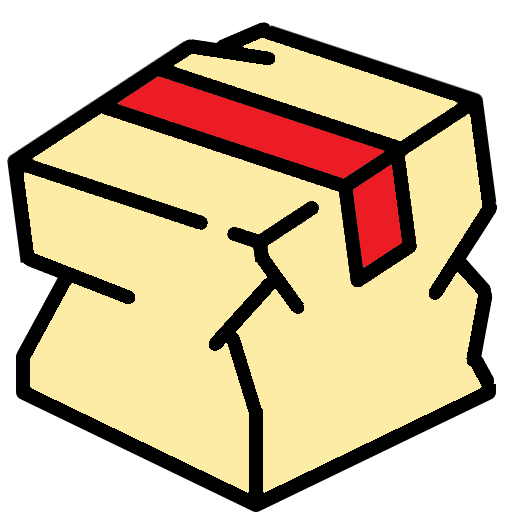

Supplement: Supplementary file 1 [file DataSheet1.ZIP › moving out game/msc.-project-nikki-bouman-7-tutorial/images/broken_heavy_box_human.png]

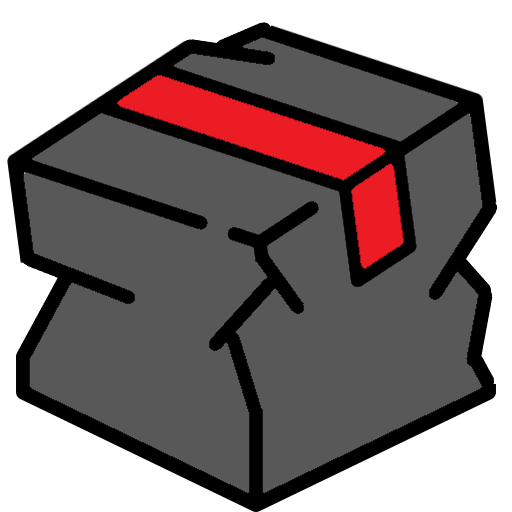

Supplement: Supplementary file 1 [file DataSheet1.ZIP › moving out game/msc.-project-nikki-bouman-7-tutorial/images/broken_heavy_box_robot.png]

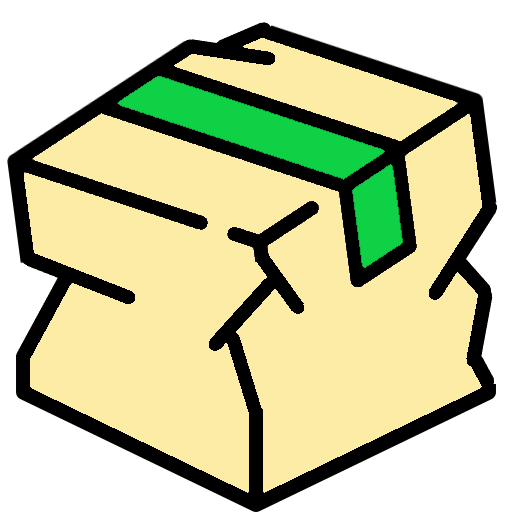

Supplement: Supplementary file 1 [file DataSheet1.ZIP › moving out game/msc.-project-nikki-bouman-7-tutorial/images/broken_light_box_human.png]

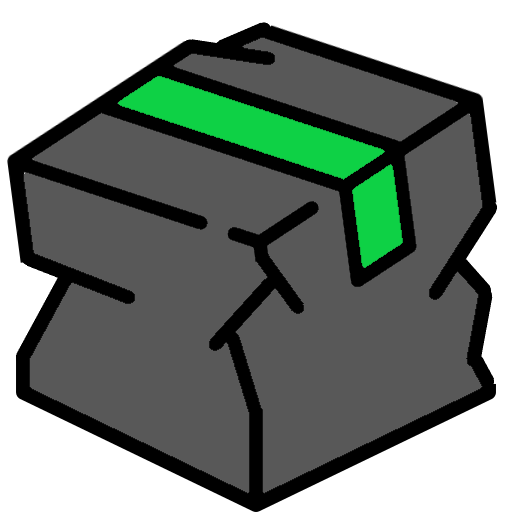

Supplement: Supplementary file 1 [file DataSheet1.ZIP › moving out game/msc.-project-nikki-bouman-7-tutorial/images/broken_light_box_robot.png]

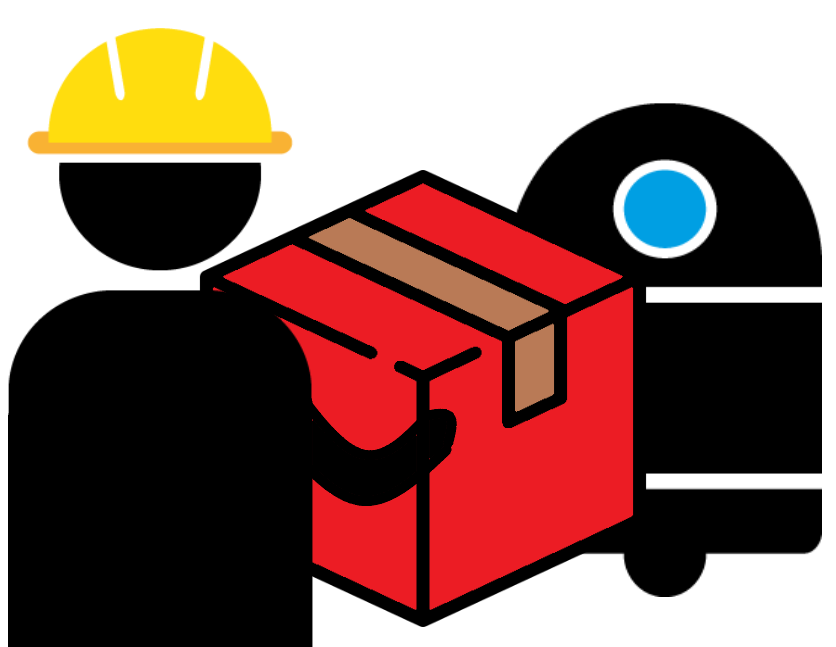

Supplement: Supplementary file 1 [file DataSheet1.ZIP › moving out game/msc.-project-nikki-bouman-7-tutorial/images/carry_heavy_box_together.png]

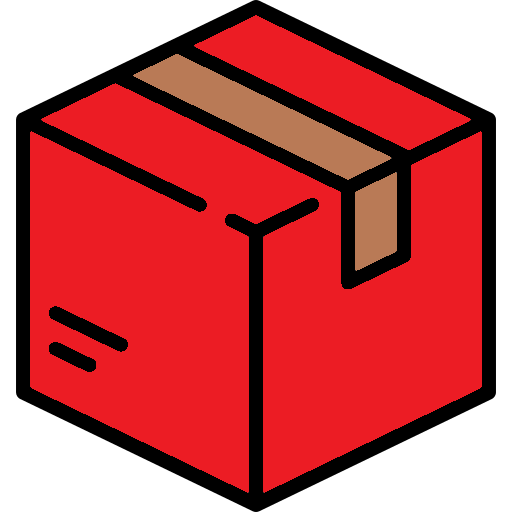

Supplement: Supplementary file 1 [file DataSheet1.ZIP › moving out game/msc.-project-nikki-bouman-7-tutorial/images/heavy_box.png]

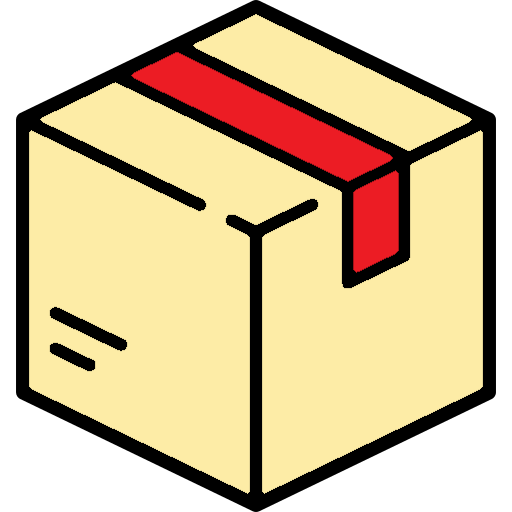

Supplement: Supplementary file 1 [file DataSheet1.ZIP › moving out game/msc.-project-nikki-bouman-7-tutorial/images/heavy_box_human.png]

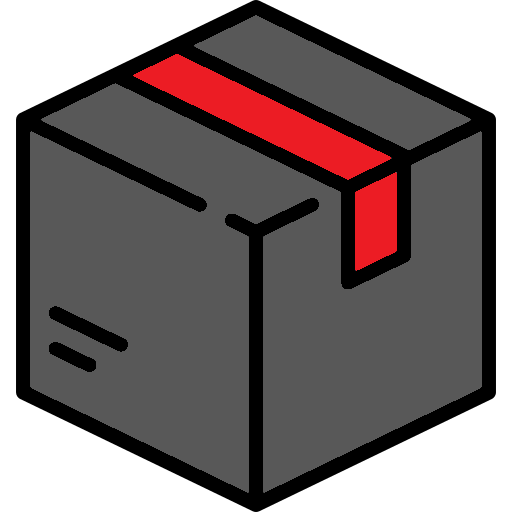

Supplement: Supplementary file 1 [file DataSheet1.ZIP › moving out game/msc.-project-nikki-bouman-7-tutorial/images/heavy_box_robot.png]

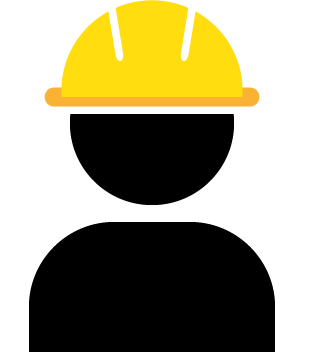

Supplement: Supplementary file 1 [file DataSheet1.ZIP › moving out game/msc.-project-nikki-bouman-7-tutorial/images/human.png]

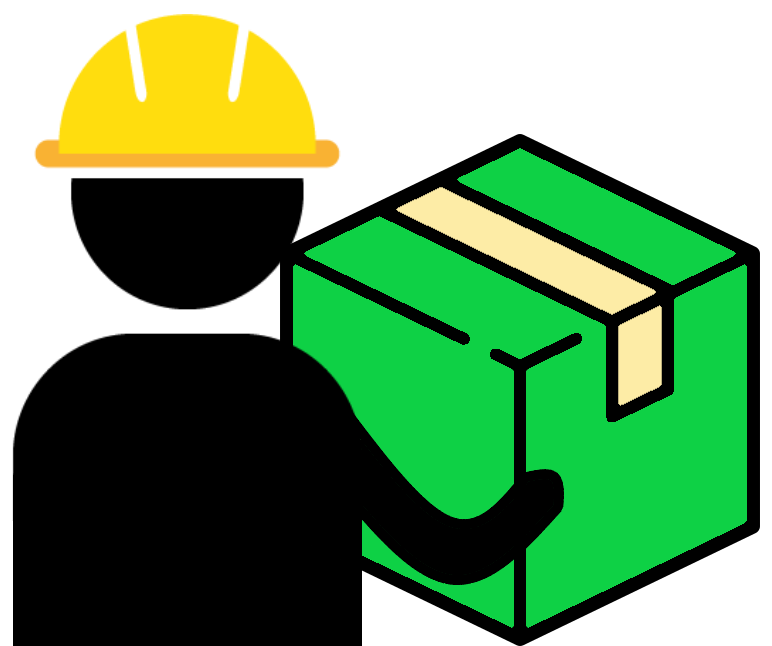

Supplement: Supplementary file 1 [file DataSheet1.ZIP › moving out game/msc.-project-nikki-bouman-7-tutorial/images/human_carry_light_box.png]

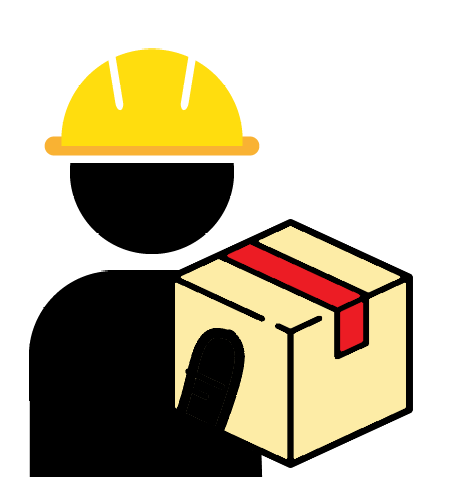

Supplement: Supplementary file 1 [file DataSheet1.ZIP › moving out game/msc.-project-nikki-bouman-7-tutorial/images/human_heavy_box_human.png]

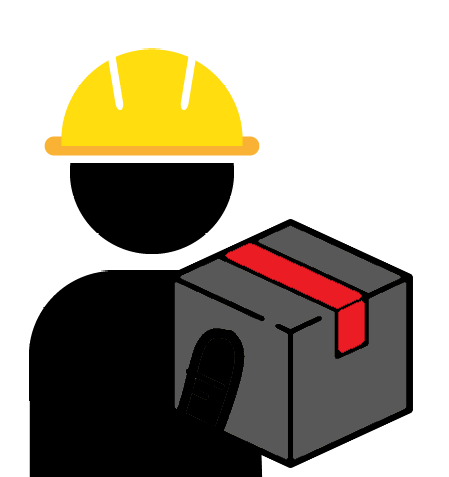

Supplement: Supplementary file 1 [file DataSheet1.ZIP › moving out game/msc.-project-nikki-bouman-7-tutorial/images/human_heavy_box_robot.png]

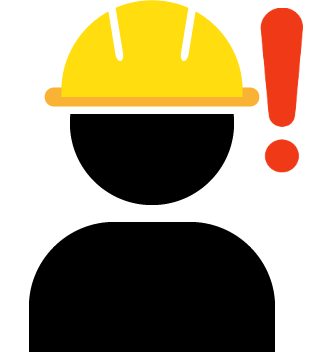

Supplement: Supplementary file 1 [file DataSheet1.ZIP › moving out game/msc.-project-nikki-bouman-7-tutorial/images/human_help.png]

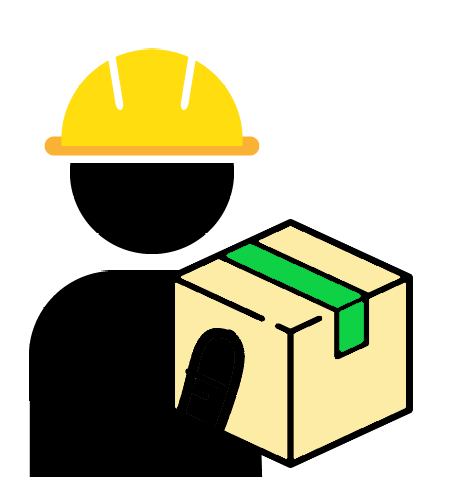

Supplement: Supplementary file 1 [file DataSheet1.ZIP › moving out game/msc.-project-nikki-bouman-7-tutorial/images/human_light_box_human.png]

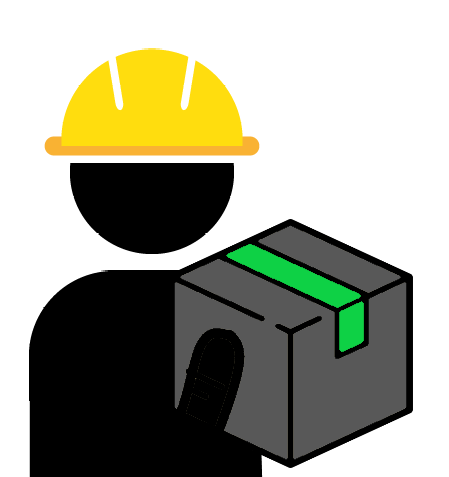

Supplement: Supplementary file 1 [file DataSheet1.ZIP › moving out game/msc.-project-nikki-bouman-7-tutorial/images/human_light_box_robot.png]

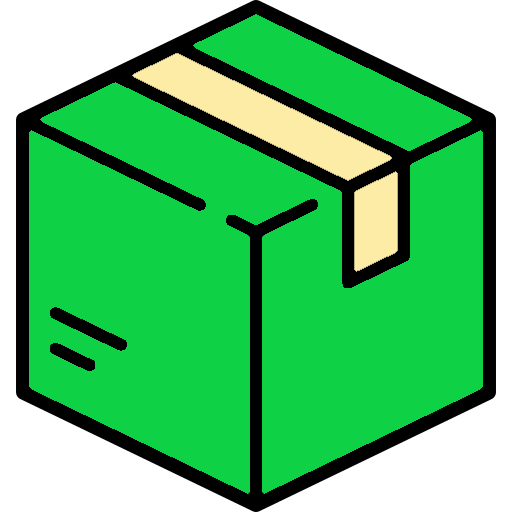

Supplement: Supplementary file 1 [file DataSheet1.ZIP › moving out game/msc.-project-nikki-bouman-7-tutorial/images/light_box.png]

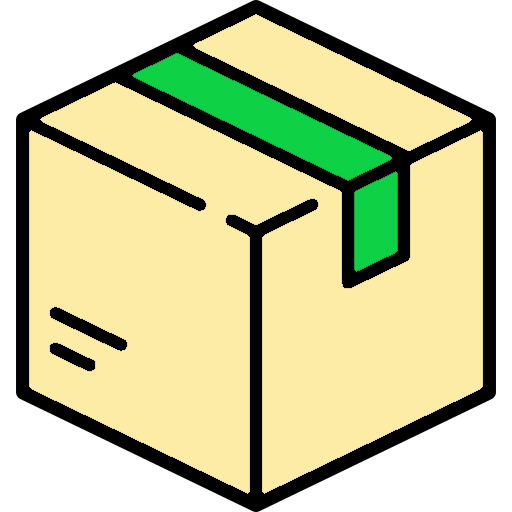

Supplement: Supplementary file 1 [file DataSheet1.ZIP › moving out game/msc.-project-nikki-bouman-7-tutorial/images/light_box_human.png]

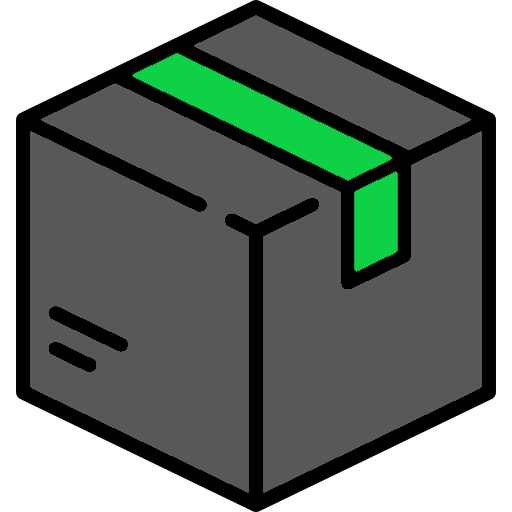

Supplement: Supplementary file 1 [file DataSheet1.ZIP › moving out game/msc.-project-nikki-bouman-7-tutorial/images/light_box_robot.png]

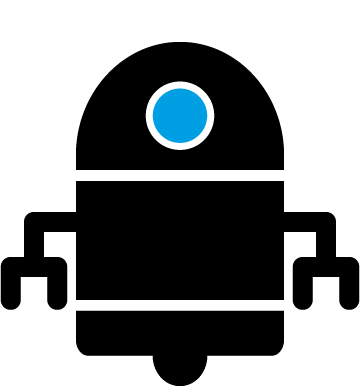

Supplement: Supplementary file 1 [file DataSheet1.ZIP › moving out game/msc.-project-nikki-bouman-7-tutorial/images/robot.png]

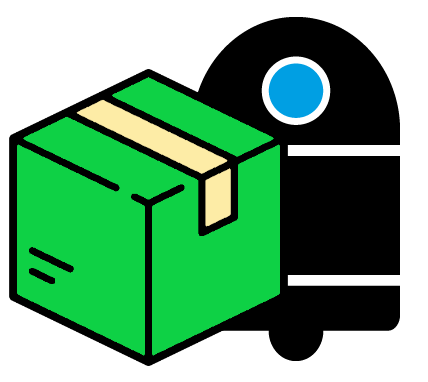

Supplement: Supplementary file 1 [file DataSheet1.ZIP › moving out game/msc.-project-nikki-bouman-7-tutorial/images/robot_carry_light_box.png]

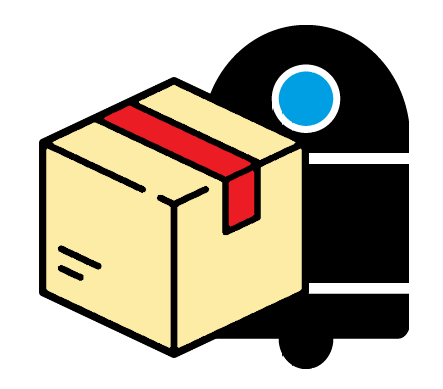

Supplement: Supplementary file 1 [file DataSheet1.ZIP › moving out game/msc.-project-nikki-bouman-7-tutorial/images/robot_heavy_box_human.png]

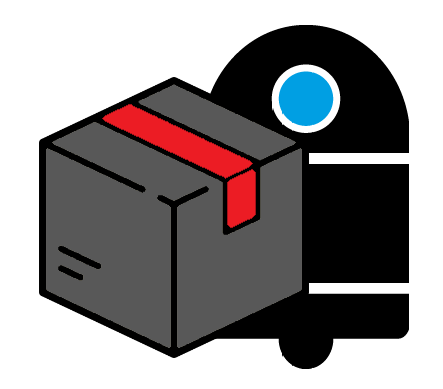

Supplement: Supplementary file 1 [file DataSheet1.ZIP › moving out game/msc.-project-nikki-bouman-7-tutorial/images/robot_heavy_box_robot.png]

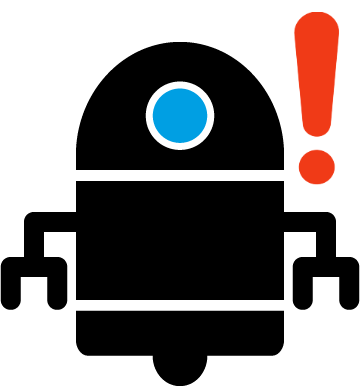

Supplement: Supplementary file 1 [file DataSheet1.ZIP › moving out game/msc.-project-nikki-bouman-7-tutorial/images/robot_help.png]

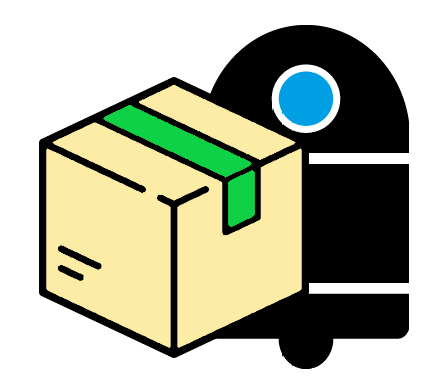

Supplement: Supplementary file 1 [file DataSheet1.ZIP › moving out game/msc.-project-nikki-bouman-7-tutorial/images/robot_light_box_human.png]

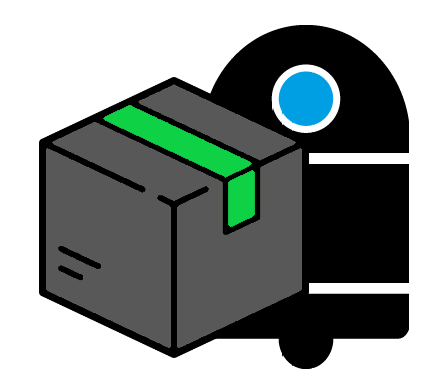

Supplement: Supplementary file 1 [file DataSheet1.ZIP › moving out game/msc.-project-nikki-bouman-7-tutorial/images/robot_light_box_robot.png]

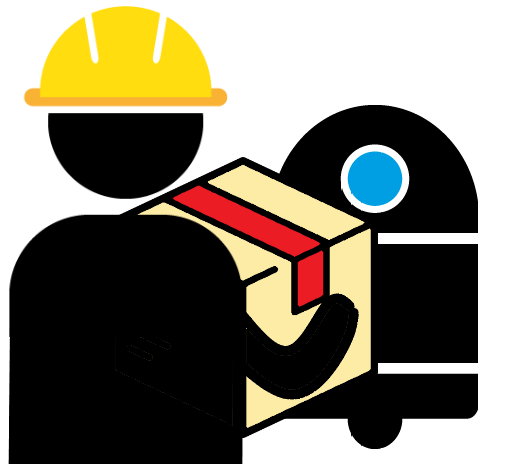

Supplement: Supplementary file 1 [file DataSheet1.ZIP › moving out game/msc.-project-nikki-bouman-7-tutorial/images/together_heavy_box_human.png]

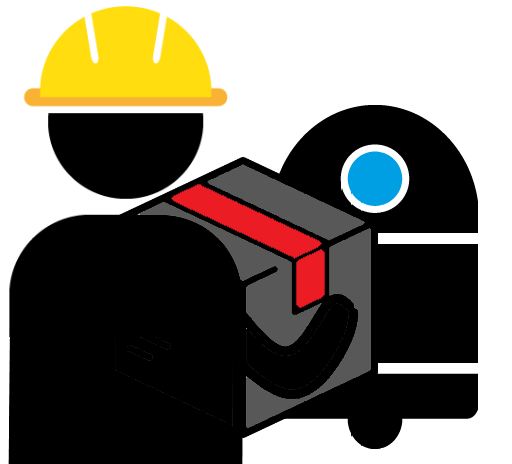

Supplement: Supplementary file 1 [file DataSheet1.ZIP › moving out game/msc.-project-nikki-bouman-7-tutorial/images/together_heavy_box_robot.png]

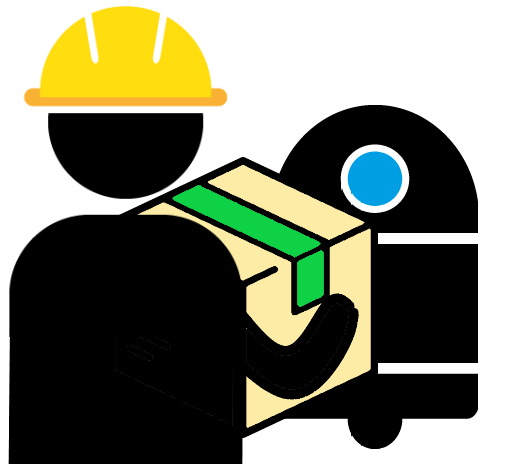

Supplement: Supplementary file 1 [file DataSheet1.ZIP › moving out game/msc.-project-nikki-bouman-7-tutorial/images/together_light_box_human.png]

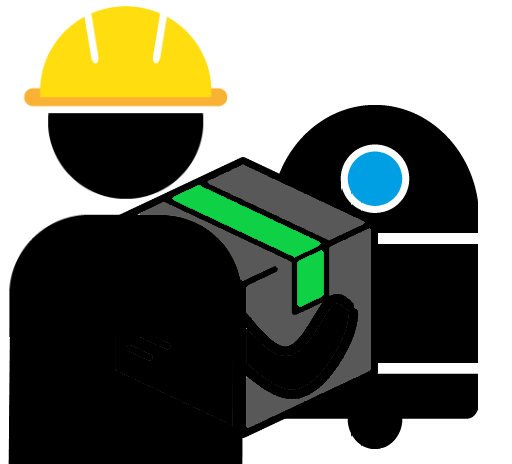

Supplement: Supplementary file 1 [file DataSheet1.ZIP › moving out game/msc.-project-nikki-bouman-7-tutorial/images/together_light_box_robot.png]

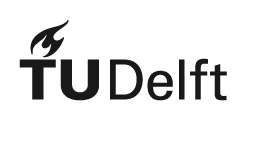

Supplement: Supplementary file 1 [file DataSheet1.ZIP › moving out game/msc.-project-nikki-bouman-7-tutorial/images/TU_logo.png]

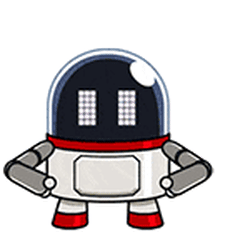

Supplement: Supplementary file 1 [file DataSheet1.ZIP › moving out game/msc.-project-nikki-bouman-7-tutorial/moving_out/case/gui/static/images/agent.gif]

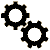

Supplement: Supplementary file 1 [file DataSheet1.ZIP › moving out game/msc.-project-nikki-bouman-7-tutorial/moving_out/case/gui/static/images/busy_black_small.gif]

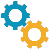

Supplement: Supplementary file 1 [file DataSheet1.ZIP › moving out game/msc.-project-nikki-bouman-7-tutorial/moving_out/case/gui/static/images/busy_matrx_small.gif]

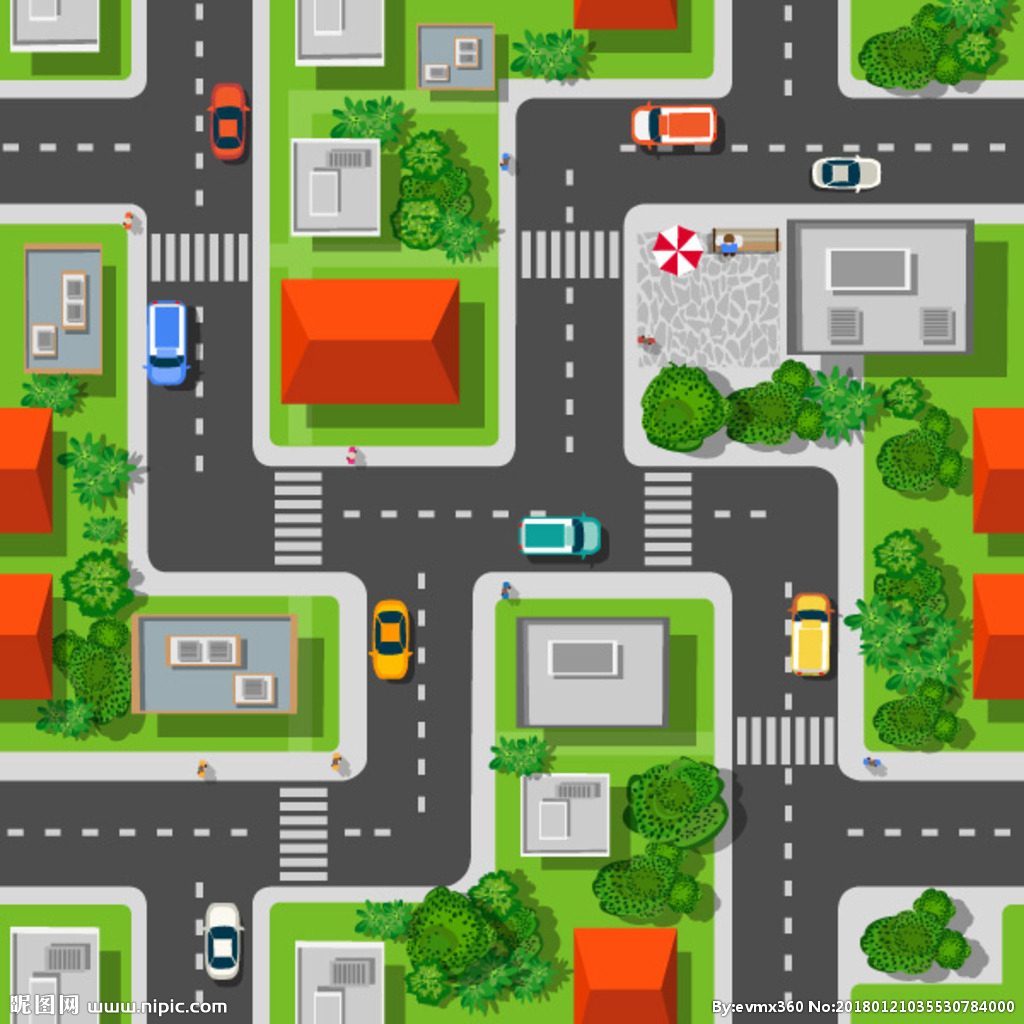

Supplement: Supplementary file 1 [file DataSheet1.ZIP › moving out game/msc.-project-nikki-bouman-7-tutorial/moving_out/case/gui/static/images/city_bg_topdown.jpg]

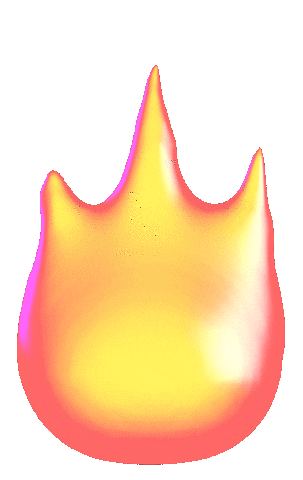

Supplement: Supplementary file 1 [file DataSheet1.ZIP › moving out game/msc.-project-nikki-bouman-7-tutorial/moving_out/case/gui/static/images/fire.gif]

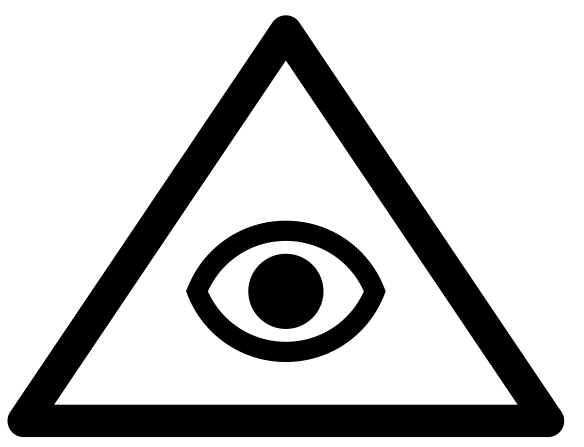

Supplement: Supplementary file 1 [file DataSheet1.ZIP › moving out game/msc.-project-nikki-bouman-7-tutorial/moving_out/case/gui/static/images/god.png]

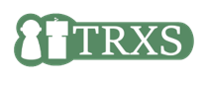

Supplement: Supplementary file 1 [file DataSheet1.ZIP › moving out game/msc.-project-nikki-bouman-7-tutorial/moving_out/case/gui/static/images/matrxs_logo.png]

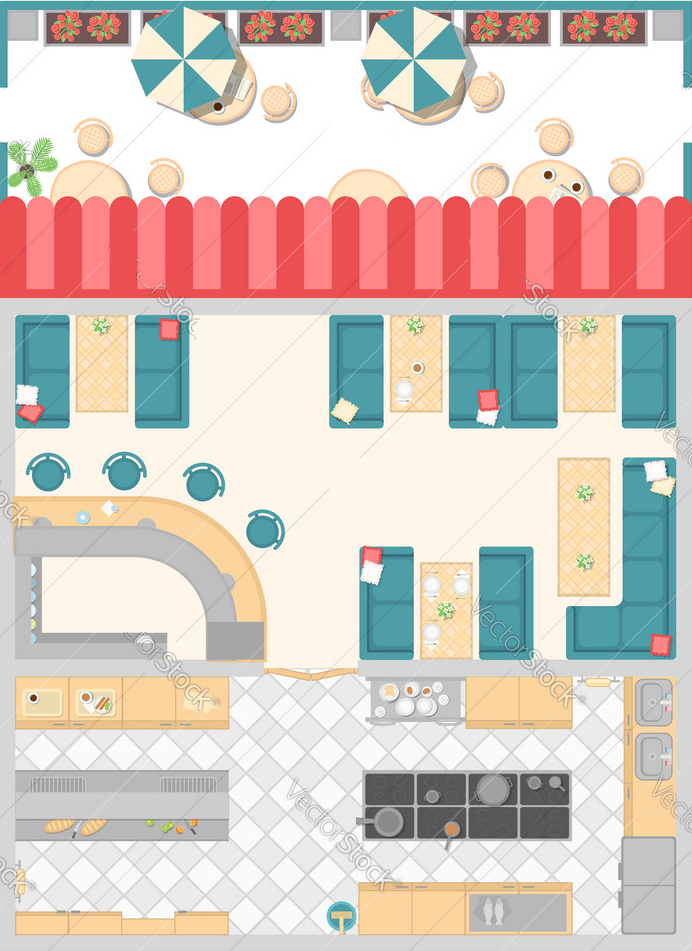

Supplement: Supplementary file 1 [file DataSheet1.ZIP › moving out game/msc.-project-nikki-bouman-7-tutorial/moving_out/case/gui/static/images/restaurant_bg.png]

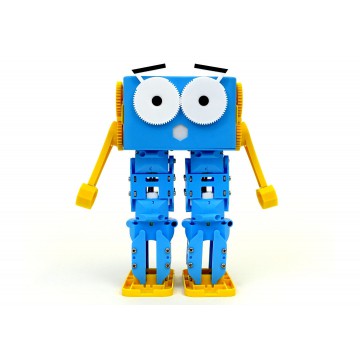

Supplement: Supplementary file 1 [file DataSheet1.ZIP › moving out game/msc.-project-nikki-bouman-7-tutorial/moving_out/case/gui/static/images/robot.jpg]

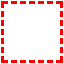

Supplement: Supplementary file 1 [file DataSheet1.ZIP › moving out game/msc.-project-nikki-bouman-7-tutorial/moving_out/case/gui/static/images/selected.png]

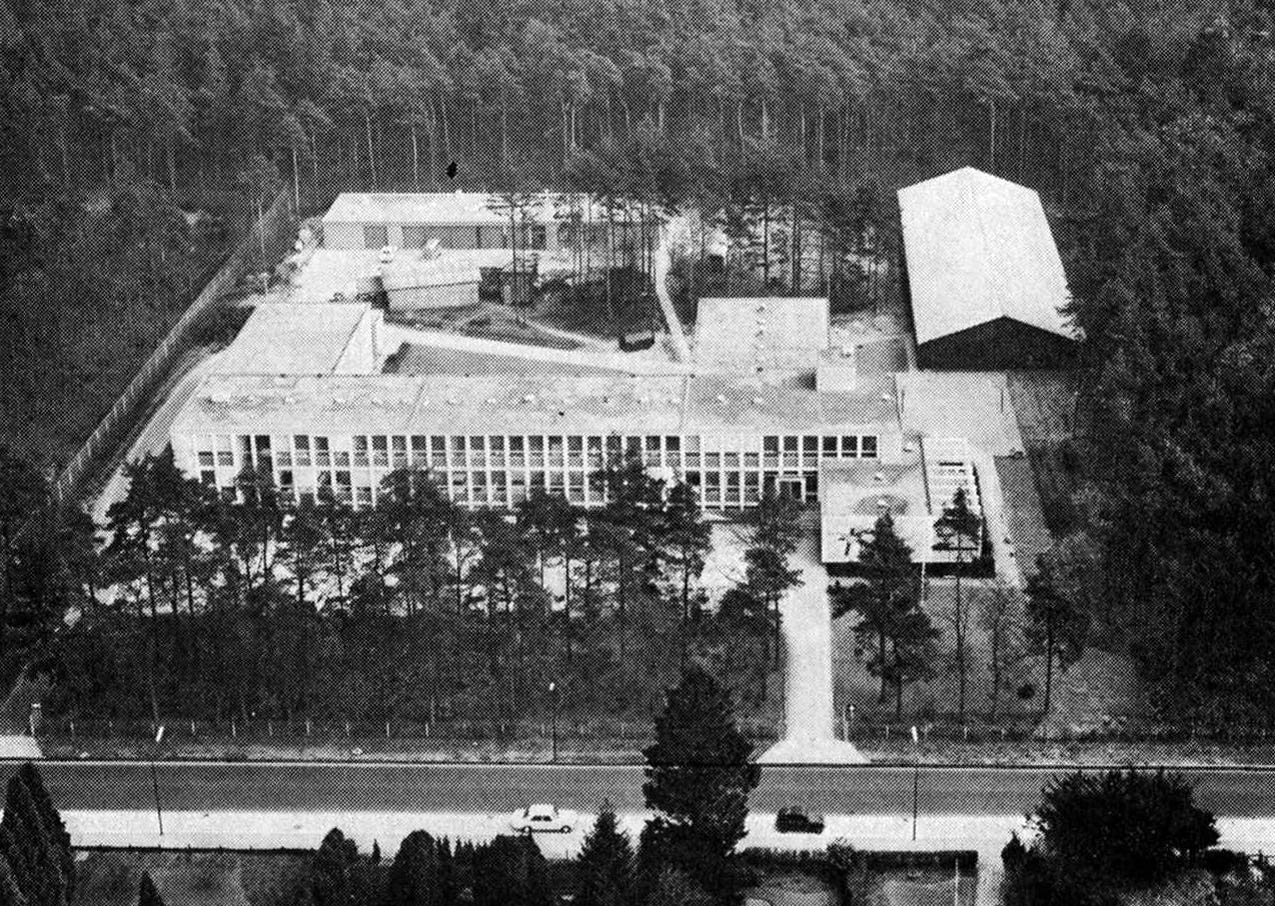

Supplement: Supplementary file 1 [file DataSheet1.ZIP › moving out game/msc.-project-nikki-bouman-7-tutorial/moving_out/case/gui/static/images/soesterberg_luchtfoto.jpg]

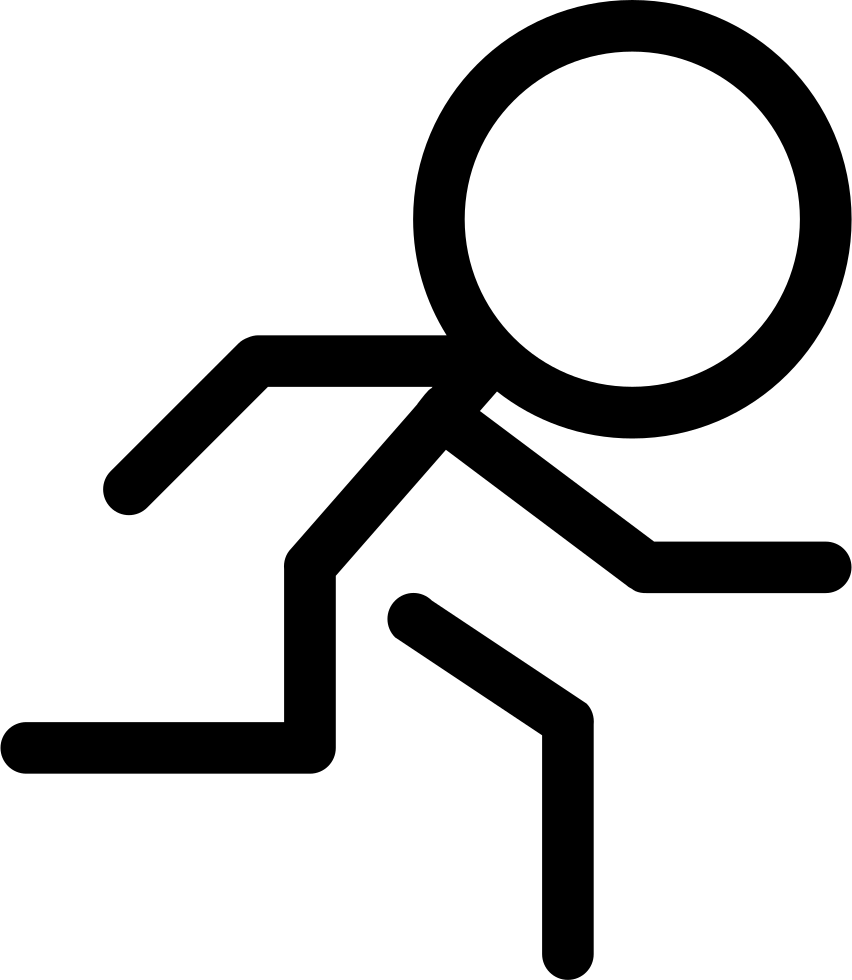

Supplement: Supplementary file 1 [file DataSheet1.ZIP › moving out game/msc.-project-nikki-bouman-7-tutorial/moving_out/case/gui/static/images/transparent.png]
